# Subclinical Atherosclerosis among HIV-Infected Adults Attending HIV/AIDS Care at Two Large Ambulatory HIV Clinics in Uganda

**DOI:** 10.1371/journal.pone.0089537

**Published:** 2014-02-28

**Authors:** Isaac Ssinabulya, James Kayima, Chris Longenecker, Mary Luwedde, Fred Semitala, Andrew Kambugu, Faith Ameda, Sam Bugeza, Grace McComsey, Juergen Freers, Damalie Nakanjako

**Affiliations:** 1 Department of Medicine, Makerere University College of Health Sciences, Kampala, Uganda; 2 Case Western Reserve University School of Medicine, Cleveland, Ohio, United States of America; 3 Makerere University Joint AIDS program, Kampala, Uganda; 4 Infectious Disease Institute, Makerere University, Kampala, Uganda; 5 Department of Radiology, Makerere University College of Health Sciences, Kampala, Uganda; University of Nebraska Medical Center, United States of America

## Abstract

**Background:**

The increased immune activation and inflammation of chronic HIV-infection and the characteristic dyslipidemias associated with HIV infection and antiretroviral therapy (ART) contribute to an increased risk of atherosclerotic vascular disease among HIV-infected adults. There is an emerging need to understand determinants of cardiovascular disease (CVD) among individuals aging with HIV in sub-Saharan Africa. We determined the prevalence of subclinical atherosclerosis [carotid intima media thickness (CIMT) ≥0.78 mm] and its correlation with traditional CVD risk factors among HIV-infected adults.

**Methods:**

In a cross-sectional study, HIV-infected adults (ART-naïve and ART-treated) were consecutively selected from patients' enrollment registers at two large HIV clinics at Mulago Hospital, Kampala, Uganda. We measured traditional CVD risk factors including age, biophysical profile, fasting blood sugar and serum lipid profile as well as biomarkers of inflammation. High resolution ultrasound was used to measure common carotid CIMT.

**Results:**

Of 245 patients, Median age [Interquartile range (IQR)] 37 years (31–43), 168 (69%) were females; and 100 (41%) were ART-treated for at least 7 years. Overall, 34/186 (18%) had subclinical atherosclerosis; of whom 15/108 (14%) were ART-naïve whereas 19/78 (24%) were ART-treated. Independent predictors of subclinical atherosclerosis included age [odds ratio (OR) 1.83 per 5-year increase in age; 95% confidence interval (CI) 1.24–2.69; p = 0.002], body mass index (BMI); OR 1.15; CI 1.01–1.31; p = 0.041 and high low density lipoprotein (LDL) [OR 2.99; CI 1.02–8.78, p = 0.046]. High sensitivity C-reactive protein (hsCRP) was positively correlated with traditional cardio-metabolic risk factors including waist circumference (r = 0.127, p = 0.05), triglycerides (r = 0.19, p = 0.003) and Total Cholesterol: High Density Lipoprotein ratio (TC:LDL) (r = 0.225, p<0.001).

**Conclusion:**

The prevalence of subclinical atherosclerosis was 18% among HIV-infected adults in Uganda. Traditional CVD risk factors were associated with subclinical atherosclerosis. We recommend routine assessment of traditional CVD risk factors within HIV care and treatment programs in sub-Saharan Africa.

## Introduction

The chronic inflammation and immune activation associated with HIV-infection and the dyslipidemia associated with Antiretroviral Therapy (ART) contribute to increased risk of atherosclerotic vascular disease among HIV-infected adults relative to the general population [1], [2]. HIV-infected adults appear to have a significantly greater risk of myocardial infarction and coronary artery disease relative to age-matched HIV-negative individuals [3], [4], [5], [6]. With the increasing life expectancy of HIV-infected adults receiving ART, cardiovascular disease has emerged as an important concern among individuals aging with HIV/AIDS [7], [8], [9]. With the scale up of life-saving antiretroviral therapy in sub-Saharan Africa (SSA), there is a growing population of individuals aging with HIV [10], [11], thus the need to understand predictors of cardiovascular disease (CVD) to inform management of CVD within HIV care and treatment programs.

HIV is associated with dyslipidemia, namely, hypocholesterolemia, hypertriglyceridemia, and low levels of both low density lipoproteins (LDL) and high density lipoproteins (HDL) [12], [13]. Whereas elevated serum lipids increase CVD risk, a low HDL contributes to the elevated risk of coronary artery disease (CAD) in HIV patients [14], [15]. In addition, ART is associated with dyslipidemia, which tends to be worse with many protease inhibitor (PI) -containing regimens [16]. Similarly, HIV-associated immune activation and inflammation are suggested to contribute to accelerated atherosclerosis and subsequently increased CVD risk among adults living with HIV/AIDS [17], [18], [19]. There are limited data on CVD and its risk factors within HIV treatment programs in SSA. There is thus a need to understand the relationship of traditional versus non-traditional risk factors with CVD among aging HIV-infected adults in this context.

Given the morbidity and mortality associated with overt CVD [3], [4], [5], [6], early identification and management of subclinical disease is desirable before complications of overt CVD develop. Measurements of carotid intima media thickness (CIMT) have previously been correlated with the extent of coronary atherosclerosis [20], [21], [22], and increases in CIMT are predictive of future CVD events [22], [23]. In this study, we measured CIMT to determine the prevalence of subclinical atherosclerosis and associated factors to inform prevention, early detection and prompt management of CVD within HIV treatment programs in SSA. We also examined the association of inflammatory markers such as high sensitivity CRP (hsCRP) with CIMT and traditional CVD risk factors in order to contribute to the development of clinical and diagnostic algorithms for CVD in settings where CIMT measurements might not be feasible in resource-limited settings.

## Methods

### Study setting

This study was conducted among HIV-infected adults enrolled at two large ambulatory HIV clinics at Mulago National referral hospital. The Makerere Joint AIDS Program immune suppression (MJAP-ISS) clinic enrolls ART-naïve HIV-infected adults that are followed up and initiated on ART according to the 2012 national guidelines of ART initiation at CD4 counts<350 cells/UL. In addition, the Infectious Diseases Institute (IDI), has an HIV treatment cohort that has received ART for seven years, having initiated ART at CD4 counts ≤200 cells/UL in 2004–2005, according to the Uganda national guidelines for ART initiation at the time. Drugs were provided through the Global Fund (a generic combined formulation of stavudine [d4T], lamivudine [3TC], and nevirapine [NVP] and the US President's Emergency Plan for AIDS Relief (a combined formulation of zidovudine [ZDV] and 3TC plus efavirenz [EFZ] or NVP). Patients with toxicity to ZDV were changed to tenofovir [TDF]; as described in previous studies [24], [25]. Protease inhibitor-containing regimens were reserved for second-line therapy if patients failed their first-line regimen.

### Study design and participants

In a cross-sectional study, HIV-infected adults ≥18 years were consecutively selected from the patient registers of ART-naive adults enrolled at MJAP-ISS clinic between February to October 2012, and the ART-treated adults in the HIV treatment cohort with HIV viral loads <400 copies/ml for at least 6 months prior to the study. Patients with confirmed CVD (stroke, myocardial infarction, and/or peripheral vascular disease), malignancy and active infection (as per the attending physicians at MJAP-ISS/IDI and medical records), were excluded. In addition, we excluded patients receiving glucocorticoids, growth hormone or other anabolic agents within the past 6 months. All eligible patients provided written informed consent to participate in the study. Scientific and ethical approvals were obtained from Makerere University School of Medicine Research and Ethics Committee, and the study was conducted according to the Declaration of Helsinki on research involving human subjects.

### Clinical assessment

Upon written informed consent and using a pre-coded and pre-tested questionnaire as a study instrument, all participants received a detailed clinical assessment to evaluate the relevant history of CVD risk factors including: lifestyle characteristics (exercise, diet, history of smoking and alcohol use), family history of CVD (hypertension, stroke, myocardial infarction and sudden death) as well as any concurrent medication that might affect CVD risk [anti-hypertensive drugs, non-steroidal anti-inflammatory drugs (NSAIDs), and lipid lowering drugs]. In addition, biophysical profile was measured by obtaining the weight in kilograms (Kg) and height in meters (m) to calculate body mass index (BMI), Kg/m^2^, waist circumference, hip circumference, waist-hip ratio and blood pressure (BP) using an automated blood pressure cuff (OMRON). We also evaluated HIV-specific risk factors such as ART status and stage of HIV disease using WHO clinical stage and CD4 count.

### Laboratory tests

A total of 10 mls of blood samples were obtained using aseptic techniques; 2 mls in EDTA tubes for hematology tests [Complete blood count and Erythrocyte Sedimentation Rate at (ESR)] and 3 mls in a general vacutainer for serum lipid profiles at the Uganda Heart Institute laboratory. Fasting blood sugar was measured using a glucometer (CONTOUR^R^TS). Blood for inflammatory markers was centrifuged for 10 minutes at 1,000×g and the serum was stored at <−70°C at the Makerere University College of Health Sciences immunology laboratory until batch analysis was done. High sensitivity C-reactive protein (hsCRP) was measured by standard ELISA technique using commercial cytokine ELISA kits from ABNOVA cat. KA0238.

### CIMT Measurement

This was done by a senior radiologist with experience in vascular imaging at Mulago National Referral Hospital, using carotid Doppler imaging. A linear array transducer with a frequency of 7–14 MHz (PHILIPS HD7) was used to obtain images of the distal 1 cm of the common carotid artery and the carotid bulb. The right and left carotid arteries were studied with the head in the midline position and tilted upward. The probe was adjusted to obtain the near and far walls in a parallel orientation and then positioned to obtain the maximal luminal diameter in the longitudinal plane. Images were recorded digitally for subsequent offline analysis using multiple manual caliper measurements. Three images of the common carotid artery were obtained following the American Society of Echo guidelines at 3 angles (anterior, lateral and posterior) bilaterally along with bilateral images of the carotid bulb at the optimum angle of insonation [26]. A mean value for each segment was obtained and the average of all measurements reported as overall CIMT for the common carotid artery. Subclinical atherosclerosis was defined using a more aggressive cut-off value of CIMT (≥0.78 mm), based on the prior observation that on average, a healthy adult reaches a CIMT of 0.78 mm at the age of 76 years [27]. The oldest participant in our study was 74 years old and >95% of participants were below 60 years old, thus justifying this lower cut-off as clearly abnormal.

### Data analysis

Data was entered in EPIDATA version 3.1 and exported to STATA 12 for analysis. Subclinical atherosclerosis, defined as CIMT ≥0.78 mm, was the primary outcome. Predictors of subclinical atherosclerosis were determined using bivariate and multivariate logistic regression. Independent predictors of subclinical cardiovascular disease such as; biophysical measurements, traditional CVD risk factors and the non-traditional CVD risk factors in HIV-infected individuals such as stage of HIV disease and ART status were analyzed. Factors with a p-value of <0.2 at bivariate analysis were included in a stepwise backward model. Subclinical atherosclerosis was also compared across Framingham risk score categories. We also determined the correlation of hsCRP (as a continuous variable) with CIMT and traditional CVD risk factors. A p-value <0.05 was considered statistically significant.

## Results

### Characteristics of study patients

**Figure 1:**
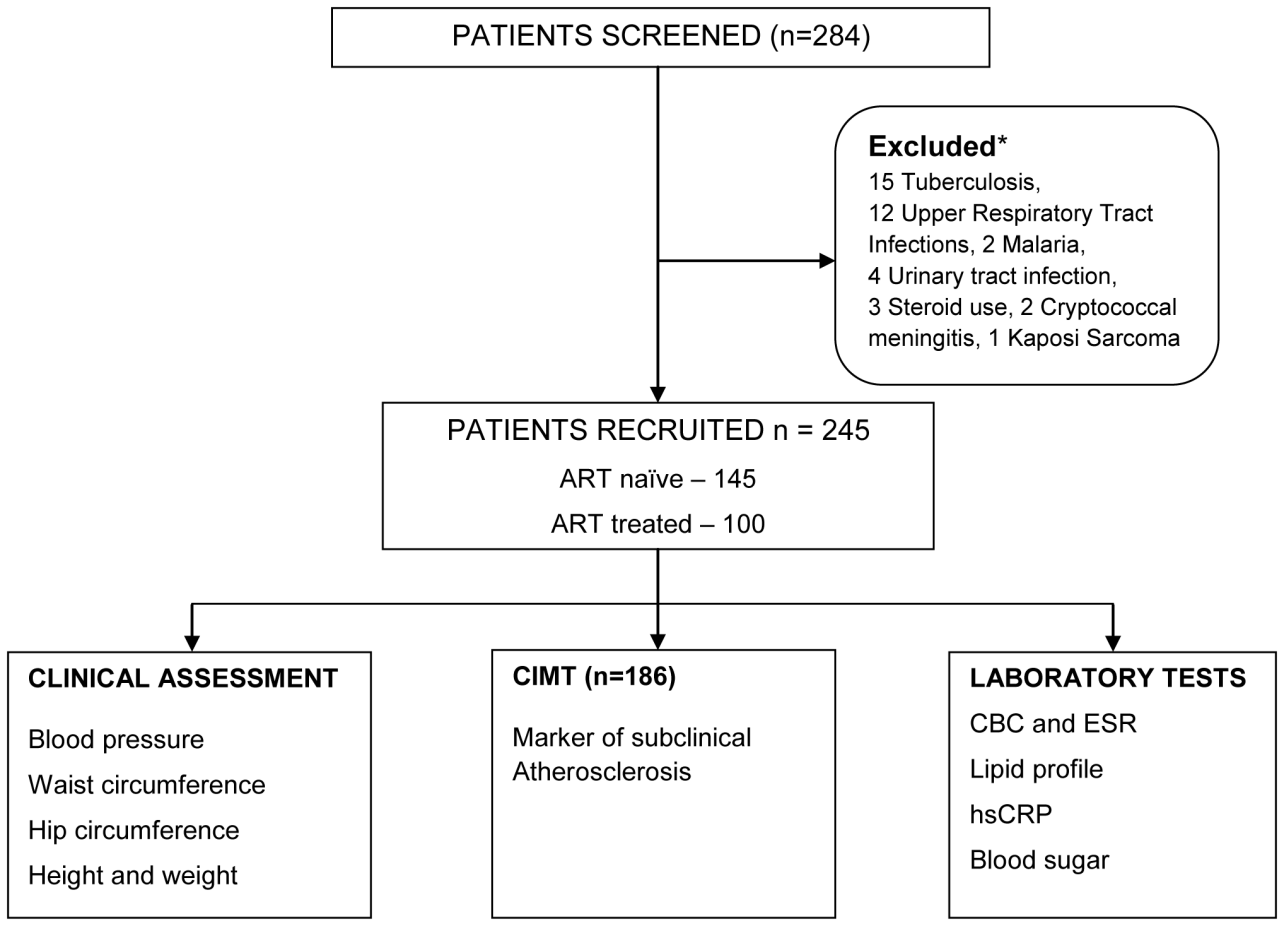
Flow diagram showing HIV-infected adults that participated in the study. * 36 patients with confirmed active disease and 3 patients receiving steroid therapy were excluded. ART: Anti retroviral therapy, CIMT: Carotid intimal medial thickness, CBC: Complete blood count, ESR: Erythrocyte sedimentation rate, hsCRP: high sensitivity C-reactive protein.

Between February and October 2012, 245 HIV-infected adults were evaluated for CVD risk. One-hundred and forty-five (59%) were ART-naïve patients and 100 (41%) had received suppressive ART for median of seven years (Figure 1). Of those on ART, 14% were on a second-line PI containing regimen. The median [inter-quartile range, (IQR)] age was 37 (31–43) years, 168 (69%) were female and 124 (51%) had no higher than primary school education. A majority of the patients 132 (54%) were in WHO stage III and IV. Among the ART-treated adults, 86% were receiving NNRTI-based first-line regimen and only 14 patients were receiving a second-line PI-containing regimen (Table 1).

**Table 1:** Characteristics of HIV infected adults attending two large ambulatory HIV clinics in Uganda.

Variable	All study participants (n = 245)	Participants with CIMT (n = 186)	
		Subclinical atherosclerosis CIMT≥0.78 (n = 34)	Normal CIMT<0.78 (n = 152)
Age, years median(IQR)	37 (31–37)	46 (37–52)	36 (30–42)
Female gender (%)	168 (69)	19 (56)	109 (72)
**Marital status**			
Not married (%)	111 (45)	13 (38)	72 (47)
Married/cohabiting (%)	134 (55)	21 (62)	80 (53)
**Education**			
Primary or no education (%)	124 (51)	16 (47)	77 (51)
Secondary and above (%)	121 (49)	18 (53)	75 (49)
**Occupation**			
Unemployed (%)	60 (24)	7 (21)	31 (20)
Employed (%)	185 (76)	27 (79)	121 (80)
**WHO stage**			
Stage I & II (%)	113 (46)	13 (38)	73 (48)
Stage III & IV (%)	132 (54)	21 (62)	79 (52)
**Use of ART (%)**	100(41)	19 (56)	59 (39)
**ART regimen**			
First line ART (%)	86 (86)	17 (50)	49 (32)
Second line ART (%)	14 (14)	2 (6)	10 (7)
**Concurrent medication**			
Septrin prophylaxis* (%)	242 (99)	34 (100)	150 (99)
Use of inflammatory drugs (NSAIDs) (%)	14 (6)	2 (6)	9 (6)
Use of antihypertensive drugs (%)	8 (3)	5 (15)	2 (1)
Use of anti-diabetic drugs (%)	3 (1)	0	2 (1)
Use lipid lowering drugs (%)	1 (<1)	0	1 (<1)
**Family history**			
Diabetes (%)	56 (23)	11 (32)	36 (24)
Hypertension (%)	94 (38)	13 (38)	60 (39)
Non traumatic stroke (%)	22 (9)	6 (18)	12 (8)
Sudden death before 50 years (%)	12 (5)	3 (9)	5 (3)
**Social history**			
Active smoking (%)	12 (5)	4 (12)	4 (3)
Passive smoker (%)	53 (22)	8 (24)	38 (25)
Alcohol use (%)	98 (40)	16 (47)	54 (36)
**Biophysical Profile (median, IQR)**			
Body mass index (Kg/m^2^)	21.5 (19.8–23.8)	22.7 (20.8–26.0)	21.6 (20.1–23.8)
Waist circumference (cm)	79 (73–86)	86 (79–94)	78 (73–85)
Hip circumference (cm)	94 (89–100)	99 (94–108)	94 (88–99)
Waist – Hip ratio	0.83 (0.78–.090)	0.86 (0.80–0.93)	0.84 (0.78–0.90)
Systolic BP (mmHg)	120 (110–131)	128 (115–144)	120 (110–130)
Diastolic BP (mmHg)	77 (70–84)	81 (71–89)	76 (69–82)
Hypertension (BP >140/90)	47 (19.4%)	12 (35.3%)	24 (16.0%)
**Laboratory measurements (median, IQR)**			
Nadir CD4 count (cell/cm^3^)	124 (42–195)	108 (23–177)	130 (36–199)
Total white cell count (x10^9^/L)	3.71 (3.15–4.63)	3.76 (3.30–4.58)	3.79 (3.22–4.74)
Hemoglobin (g/dL)	13.2 (11.9–14.3)	13.9 (12.8–15.0)	13.1 (11.9–14.4)
Fasting blood sugar (mg/dL)	94 (85–103)	90 (85–105)	94 (83–104)
Total cholesterol (mmol/l)	3.9 (3.4–4.8)	4.5 (3.5–4.9)	3.9 (3.3–4.7)
LDL- cholesterol (mmol/l)	2.0 (1.50–2.60)	2.41 (1.90–2.90)	1.93 (1.50–2.48)
HDL- cholesterol (mmol/l)	0.90 (0.70–1.30)	0.94 (0.65–1.46)	0.90 (0.74–1.10)
non HDL (mmol/l)	2.90 (2.40–3.62)	3.30 (2.74–3.64)	2.83 (2.30–3.70)
Triglyceride (mmol/l)	1.10 (0.82–1.63)	1.53 (0.92–2.12)	1.04 (0.78–1.50)
TCHDL ratio	4.26 (3.22–5.43)	4.70 (3.32–5.71)	4.23 (3.35–5.17)
ESR (mm/hr)	10 (1–24)	2 (1–11)	10 (1–24)
hsCRP (mg/dl)	3.50 (1.80–7.70)	2.7 (1.65–7.35)	3.45 (1.80–7.00)

CIMT: Carotid Intima Media Thickness, IQR: Inter quartile range, WHO: World Health Organization, ART: Anti-Retroviral Therapy, NSAID: Non-Steroid Anti Inflammatory Drugs e.g. Ibuprofen, HDL: High Density Lipoprotein, LDL: Low Density Lipoprotein, TC-HDL: Total Cholesterol- High Density Lipoprotein ratio, hsCRP: high sensitivity C-reactive protein, ESR: Erythrocyte sedimentation rate

*Three patients were taking Dapsone as prophylaxis for opportunistic infections

### Subclinical atherosclerosis among HIV-infected adults

Carotid ultrasound imaging was performed in 186 of the study subjects. Fifty-nine subjects were not imaged due to logistical difficulties in arranging the ultrasound examination. Baseline characteristics of these patients were not different from those who had carotid imaging. Subclinical atherosclerosis was observed in (34/186) 18% of patients, 19 (56%) of whom were receiving ART. Only 2 of the 14 receiving a PI-containing regimen as second-line therapy had CIMT of ≥0.78 mm. Factors independently associated with subclinical atherosclerosis (Table 2) included every five-year increase in age, [odds ratio (OR) 1.83; 95% confidence interval (CI) 1.24–2.69; p = 0.002], every unit increase in BMI [OR 1.15; CI 1.01–1.31; p = 0.041] and high LDL-Cholesterol [OR 2.99; CI 1.02–8.78; p = 0.046]. CD4 count, ART use in general, and PI use specifically, were not associated with subclinical atherosclerosis in our models.

**Table 2:** Logistic regression for factors associated with subclinical atherosclerosis among HIV infected adults attending two large ambulatory HIV clinics in Uganda.

	BI-VARIATE ANALYSIS		MULTI VARIATE ANALYSIS	
Variable	Crude odds ratio (95%CI)	P – value	Adjusted odds ratio (95%CI)	P – value
**Demographic characteristics**				
Age (in 5 year increase)	1.75 (1.37–2.24)	**<0.001**	1.83 (1.24–2.69)	**0.002**
Gender				
Males	1.00		1.00	
Female	0.50 (0.23–1.07)	0.075	0.96 (0.25–3.70)	0.317
**Biophysical profile**				
Body Mass Index (Kg/m^2^)	1.09 (1.00–1.19)	**0.039**	1.15 (1.01–1.31)	**0.041**
Waist circumference (cm)	1.07 (1.03–1.12)	**<0.001**		
Hip circumference (cm)	1.04 (1.01–1.04)	**0.012**		
Waist-Hip ratio (0.1 increase)	1.35 (0.88–2.09)	0.173	0.69 (0.33–1.43)	0.317
Systolic BP (mmHg) (increase of 5)	1.13 (1.03–1.23)	**0.007**	1.08 (0.85–1.36)	0.532
Diastolic BP (mmHg) (increase of 5)	1.21 (1.04–1.41)	**0.015**	0.91 (0.61–1.38)	0.674
**HIV disease marker**				
CD4	1.00 (0.99–1.00)	0.333		
**Laboratory measurements**				
White Blood Cell count (per mm^3^)	0.85 (0.63–1.14)	0.259		
Haemogram (g/dl)	1.22 (1.00–1.49)	**0.043**	1.31 (0.94–1.84)	0.116
Fasting blood glucose (mg/dl)	1.00 (0.99–1.01)	0.632		
Fasting lipid profile				
Total cholesterol (mmol/l)	1.35 (0.93–1.95)	0.120	0.34 (0.12–0.94)	**0.038**
HDL cholesterol (mmol/l)	1.32 (0.88–1.96)	0.280		
LDL cholesterol (mmol/l)	1.65 (1.07–2.54)	**0.024**	2.99 (1.02–8.78)	**0.046**
NonHDL cholesterol (mmol/l)	1.32 (0.88–1.96)	0.175		
TC-HDL	1.07 (0.85–1.35)	0.557		
Triglycerides (mmol/l)	1.28 (0.93–1.76)	0.135	1.52 (0.87–2.66)	0.140
Inflammatory markers				
hsCRP (mg/L)	0.99 (0.97–1.02)	0.586		
ESR (mm/hr)	0.99 (0.96–1.01)	0.170	0.99 (0.97–1.02)	0.586
**Medical and social history**				
Use of ART				
*Yes*	1.00		1.00	
*No*	0.5 (0.23–1.06)	0.071	0.59 (0.14–2.47)	0.473
Use of hypertensive drugs				
*Yes*	1.00		1.00	
*No*	0.08 (0.01–0.42)	**0.003**	0.5 (0.23–3.52)	0.313
Family history of obesity				
*Yes*	1.00		1.00	
*No*	0.44 (0.19–0.99)	**0.05**	0.80 (0.23–2.81)	0.736
Active smoking				
Yes	1.00		1.00	
No	0.20 (0.05–0.86)	**0.03**	0.16 (0.02–1.27)	0.089
Alcohol use				
*Yes*	1.00			
*No*	0.62 (0.29–1.31)	0.212		

CIMT: Carotid Intima Media Thickness, HDL: High Density Lipoprotein, LDL: Low Density Lipoprotein, TC-HDL: Total Cholesterol- High Density Lipoprotein ratio, hsCRP: high sensitivity C - reactive protein, ESR: Erythrocyte sedimentation rate, ART: Antiretroviral therapy.

Patients with subclinical atherosclerosis had a higher median [Inter-quartile range, (IQR)] Framingham risk score compared to those with normal CIMT 7.55 (2.22–11.97) vs. 1.56 (0.68–4.31), respectively, p<0.0001; (Table 3). For every 10% increase in Framingham risk score, the odds of having an abnormal CIMT tripled; [OR 2.96; 95% CI (1.72–5.10); p<0.0001].

**Table 3:** The distribution of subclinical atherosclerosis across the Framingham risk score categories (n = 181).

Framingham risk score	Normal CIMT	Subclinical atherosclerosis (CIMT ≥0.78 mm)
Less than 10%	135 (90.6%)	22 (68.75%)
10–20%	9 (6.0%)	6 (18.75%)
More than	5 (3.4%)	4 (12.5%)
Median score (95%CI)	1.56 (0.68–4.31)	7.55 (2.22–11.97)

Note: 5 of the participants had missing variables for FRS score hence not included.

CIMT: Carotid intima media thickness, CI: Confidence interval

ESR was >20 mm/hr among 71 (32%) of HIV infected patients, of whom 13 (18%) were on ART. HsCRP was ≥3 mg/L in 130 (54%) HIV patients, of whom 56 (43%) were on ART. There was no correlation between CIMT and hsCRP (Spearman's correlation (r) = −0.051 p = 0.49). Hs-CRP was, however, positively correlated with several traditional CVD risk factors (Table 4): waist circumference (r = 0.127, p = 0.05), triglycerides (r = 0.19; p = 0.003), and TC/HDL ratio (r = 0.225; p<0.001).

**Table 4:** Spearman correlation coefficients of hsCRP concentrations with CIMT and traditional cardiovascular risk factors among HIV infected adults.

	Independent variable	Spearman's rank correlation coefficient (r)	p-value
	CIMT, mm	−0.051	0.49
**Traditional risk factors**			
	Age, years	−0.005	0.93
**Biophysical profile**	Body Mass Index, Kg/m^2^	0.048	0.46
	Waist circumference, cm	0.127	0.05
	Hip circumference, cm	0.066	0.32
	Waist-hip ratio	0.072	0.28
**Hypertension**	Systolic Blood Pressure, mmHg	0.072	0.91
	Diastolic Blood Pressure, mmHg	0.008	0.90
**Laboratory measurements**	Fasting blood glucose, mg/dl	0.095	0.16
	Total cholesterol, mmol/l	0.050	0.44
	HDL cholesterol, mmol/l	−0.159	0.014
	LDL cholesterol, mmol/l	0.066	0.32
	Triglycerides, mmol/l	0.189	0.003
	NonHDL, mmol/l	0.117	0.07
	TC:HDL	0.225	<0.001
**Other factors**			
**Inflammatory marker**	Erythrocyte Sedimentation Rate, mm/hr	0.015	0.015
**Additional blood parameters**	Total White Blood Count, x10^9^/L	0.158	0.015
	Hemoglobin concentration, g/dl	0.004	0.95
**HIV disease marker**	CD4, lymphocytes/mm3; median (IQR)	−0.024	0.71

CIMT: Carotid Intima Media Thickness, HDL: High Density Lipoprotein, LDL: Low Density Lipoprotein, TC-HDL: Total Cholesterol- High Density Lipoprotein ratio, hsCRP: high sensitivity C - reactive protein.

## Discussion

The prevalence of study-defined subclinical atherosclerosis was 18% among HIV-patients, 56% of whom were receiving ART. This is a lower prevalence compared to what has been found in studies done in developed countries [28], [29], [30]. In a cross sectional study of 187 HIV-infected patients attending clinics of Hospital Universitari de Santo Joan, Spain, [28]; a high prevalence of subclinical atherosclerosis at 65% was reported [28]. Despite a comparable cut-off of 0.80 mm, patients in the latter study were mainly males and more than 80% were cigarette smokers. Among ART-naïve HIV-infected adults attending hospitals in the Italian coordination group for the study of allergies and HIV infection, De Socio et al [29], reported subclinical atherosclerosis at 41.7% using CIMT cut-off of 0.9 mm. About half (51%) of the subjects in De Socio et al [29] study, had a history of smoking; median age was 40 years and majority of participants (79.2%) were males. It is important to note that our CIMT cut-off of 0.78 mm would have provided even higher rate of subclinical atherosclerosis in these western studies. Our study used this cut-off basing on previous findings from an observational study in the Netherlands, which demonstrated that a healthy adult reaches a CIMT of 0.78 mm at the age of 76 years [27]. In a European cohort Maggi et al [30] found 14.3% of ART naïve and 32.2% of HAART-treated patients to have a CIMT >1.00 mm. Despite a comparable age to our study patients, majority of the patients in the latter study were males (75%) and over two thirds had a history of cigarette smoking, in addition to higher rates of dyslipidemia. Even with our stringent cut-off of 0.78 mm for CIMT, our patients had lower rates of subclinical atherosclerosis despite seven year of ART. We attribute these differences to the generally low prevalence of the traditional CVD risk factors (i.e. smoking) in Uganda relative to the western cohorts [28], [29], [30], [31]. Although quantifiably smaller than other HIV-infected cohorts, our results reveal an emerging risk of future CVD among adults aging with HIV/AIDS in Uganda. This calls for longitudinal studies to understand the modifiable CVD risk factors in this population. In addition, there is need to further understand the mechanisms by which HIV directly or indirectly through inflammation, may accelerate the incidence of non-AIDS defining illnesses to inform the preventive and therapeutic interventions for people living with HIV/AIDS.

Our study shows that traditional CVD risk factors such as advancing age, obesity, and high LDL-Cholesterol were associated with subclinical atherosclerosis among patients with HIV infection in Uganda. Several studies in HIV have previously shown the association of traditional CVD risk factors and atherosclerosis as measured by CIMT [18], [29], [31], [32], [33], [34], [35]. Currier et al [35] found that Carotid IMT was significantly greater with older age, male gender, greater BMI, greater waist-to-hip ratio, increased systolic blood pressure, total cholesterol, glucose, homocysteine, regular smoking, alcohol consumption, lipodystrophy, and antiretroviral therapy. After multivariable adjustment, however, only conventional CVD risk factors were independently associated with increased CIMT in HIV-infected patients. In our study, hsCRP was not associated with subclinical atherosclerosis, despite the fact that levels were elevated (≥3 mg/L) in over half of our patients. Our results are comparable with previous reports that hsCRP did not correlate with CIMT, particularly at the Common Carotid Artery (CCA) segment, among ART-treated HIV-infected adults [19], [36]. We measured CCA IMT because it is easier to measure and results are more reproducible, relative to CIMT as measured from the internal carotid artery (ICA) [26], [37], [38]. The American Society of Echocardiography and Society of Vascular Medicine and Biology, in a report on the use of vascular ultrasound in cardiovascular risk stratification, recommended the use of the CCA segment for improved reproducibility since there was no compelling evidence to suggest that combined measurements or measurement of a specific segment is clearly superior [37].

We demonstrated a positive correlation between hsCRP and traditional CVD risk factors including waist circumference, triglycerides, total cholesterol, however there was no correlation between hsCRP and CIMT. Similarly, there are previous studies in some Western cohorts that demonstrated a positive correlation between hsCRP and traditional CVD risk factors such as LDL-cholesterol, HDL-cholesterol, BMI, waist-to-hip ratio (WHR) and smoking [39], [40], [41]. Guimaraes et al found that hsCRP levels correlated positively with waist measurement (p = 0.004), WHR (p<0.001), systolic (p = 0.05) and diastolic (p = 0.03) blood pressure, intra-abdominal fat thickness (p = 0.02), triglycerides (p = 0.001), total cholesterol (p = 0.01), and fasting glucose (p = 0.01) [41]. Similarly, in a cross sectional study of 245 HIV-infected patients, 99 (40%) had a CRP of >3 mg/L. CRP positively correlated with total cholesterol, LDL, triglycerides and negatively correlated with HDL [40].

Given the cross-sectional nature of our study we could not provide conclusive evidence about the relationship between hsCRP and CIMT since we did not actively exclude the multi-factorial causes of raised CRP within our study population such as malaria, helminthes and subclinical hepatocyte dysfunction [42]; among others. In addition, we did not have an HIV-negative control group to provide background data on hsCRP and CIMT in this community. Also, we did not measure other markers of systemic inflammation that have been shown to correlate with cardiovascular disease in HIV, such as IL-6.

### Implication of the study

Subclinical atherosclerosis was found in 18% of HIV-infected adults attending HIV care programs at Mulago hospital. Although a lower prevalence than prior studies in developed countries, this subclinical atherosclerosis is likely to lead to clinically important cardiovascular disease for these participants over time. With the global scale up of ART, more HIV-infected adults are living longer, have higher BMI, and experience more dyslipidemias. In this study, we found that these traditional cardiovascular risk factors were associated with subclinical atherosclerosis among HIV-infected adults. This implies that HIV care facilities need to be ready to screen for CVD and identify at risk patients for early intervention. This can be achieved through increasing awareness among health care providers about the need to evaluate CVD among their clients as well as provision of facilities to measure the known traditional risk factors within the HIV care programs. For example; there is a need to train all health workers in CVD assessment, having the necessary equipment to use in CVD risk assessment and detection, for example, sphygmomanometers, scales, and tape measures for measuring blood pressure, BMI, and waist circumference. There is a need to have access to laboratories that can monitor lipid profile for HIV-infected patients. In addition, the HIV care patient charts should have provisions to record these risk factors for the purpose of CVD risk modification. Similarly, ongoing counseling and education on risk reduction is required to prevent CVD among people living HIV.

### Study limitations

We recognize that the lack of an HIV-negative control population is a limitation of our study; however, this study was not designed to determine the effect of HIV on CVD risk but rather to explore the magnitude of CVD risk among adults in HIV treatment programs in the SSA context, including the risk associated with ART use. Secondly, we used hs-CRP as a single biomarker of systemic inflammation, although other markers such as IL-6 have been correlated with CVD in HIV subjects. In addition, systemic immune activation, specifically monocyte activation was not measured. Traditional CVD risk factors were strongly correlated, however, with generalized inflammation as measured by hsCRP in our study, suggesting that initial prevention strategies should target traditional CVD risk factors among adults living with HIV in Uganda. Future longitudinal cohort studies among HIV and non-HIV individuals in SSA are needed to further understand relationships between traditional and non-traditional CVD risk factors in our setting.

### Conclusion

Overall prevalence of subclinical atherosclerosis among HIV-infected adults was 18% among HIV-infected patients. Traditional CVD risk factors were associated with subclinical atherosclerosis during HIV disease. HsCRP had a positive correlation with traditional CVD risk factors. We recommend routine assessment of CVD using traditional risk factors within HIV care and treatment programs in sub-Saharan Africa.

## References

[1] Obel N, Thomsen HF, Kronborg G, Larsen CS, Hildebrandt PR, et al (2007) Ischemic heart disease in HIV-infected and HIV-uninfected individuals: a population-based cohort study. Clin Infect Dis 44: 1625–1631. 17516408 10.1086/518285

[2] Triant VA, Lee H, Hadigan C, Grinspoon SK (2007) Increased acute myocardial infarction rates and cardiovascular risk factors among patients with human immunodeficiency virus disease. J Clin Endocrinol Metab 92: 2506–2512. 17456578 10.1210/jc.2006-2190 PMC2763385

[3] Currier JS, Taylor A, Boyd F, Dezii CM, Kawabata H, et al (2003) Coronary heart disease in HIV-infected individuals. J Acquir Immune Defic Syndr 33: 506–512. 12869840 10.1097/00126334-200308010-00012

[4] Friis-Moller N, Sabin CA, Weber R, d'Arminio Monforte A, El-Sadr WM, et al (2003) Combination antiretroviral therapy and the risk of myocardial infarction. N Engl J Med 349: 1993–2003. 14627784 10.1056/NEJMoa030218

[5] Klein D, Hurley LB, Quesenberry Jr CP, Sidney S (2002) Do protease inhibitors increase the risk for coronary heart disease in patients with HIV-1 infection? J Acquir Immune Defic Syndr 30: 471–477. 12154337 10.1097/00126334-200208150-00002

[6] Vittecoq D, Escaut L, Chironi G, Teicher E, Monsuez JJ, et al (2003) Coronary heart disease in HIV-infected patients in the highly active antiretroviral treatment era. AIDS 17 Suppl 1S70–76. 12870533 10.1097/00002030-200304001-00010

[7] Gortmaker SL, Hughes M, Cervia J, Brady M, Johnson GM, et al (2001) Effect of combination therapy including protease inhibitors on mortality among children and adolescents infected with HIV-1. N Engl J Med 345: 1522–1528. 11794218 10.1056/NEJMoa011157

[8] Palella Jr FJ, Delaney KM, Moorman AC, Loveless MO, Fuhrer J, et al (1998) Declining morbidity and mortality among patients with advanced human immunodeficiency virus infection. HIV Outpatient Study Investigators. N Engl J Med 338: 853–860. 9516219 10.1056/NEJM199803263381301

[9] Sterne JA, Hernan MA, Ledergerber B, Tilling K, Weber R, et al (2005) Long-term effectiveness of potent antiretroviral therapy in preventing AIDS and death: a prospective cohort study. Lancet 366: 378–384. 16054937 10.1016/S0140-6736(05)67022-5

[10] UNAIDS Global Report (2012) Available: http://www.unaids.org/en/media/unaids/contentassets/documents/epidemiology/2012/gr2012/20121120.Accessed: 30 Sep 2013.

[11] Szadkowski L, Tseng A, Walmsley SL, Salit I, Raboud JM (2012) Short communication: effects of age on virologic suppression and CD4 cell response in HIV-positive patients initiating combination antiretroviral therapy. AIDS Res Hum Retroviruses 28: 1579–1583. 22734840 10.1089/AID.2012.0018

[12] Shor-Posner G, Basit A, Lu Y, Cabrejos C, Chang J, et al (1993) Hypocholesterolemia is associated with immune dysfunction in early human immunodeficiency virus-1 infection. Am J Med 94: 515–519. 7605397 10.1016/0002-9343(93)90087-6

[13] Riddler SA, Smit E, Cole SR, Li R, Chmiel JS, et al (2003) Impact of HIV infection and HAART on serum lipids in men. JAMA 289: 2978–2982. 12799406 10.1001/jama.289.22.2978

[14] David MH, Hornung R, Fichtenbaum CJ (2002) Ischemic cardiovascular disease in persons with human immunodeficiency virus infection. Clin Infect Dis 34: 98–102. 11731952 10.1086/324745

[15] Varriale P, Saravi G, Hernandez E, Carbon F (2004) Acute myocardial infarction in patients infected with human immunodeficiency virus. Am Heart J 147: 55–59. 14691419 10.1016/j.ahj.2003.07.007

[16] Carr A, Samaras K, Thorisdottir A, Kaufmann GR, Chisholm DJ, et al (1999) Diagnosis, prediction, and natural course of HIV-1 protease-inhibitor-associated lipodystrophy, hyperlipidaemia, and diabetes mellitus: a cohort study. Lancet 353: 2093–2099. 10382692 10.1016/S0140-6736(98)08468-2

[17] Triant VA, Meigs JB, Grinspoon SK (2009) Association of C-reactive protein and HIV infection with acute myocardial infarction. J Acquir Immune Defic Syndr 51: 268–273. 19387353 10.1097/QAI.0b013e3181a9992c PMC2763381

[18] Ross AC, O'Riordan MA, Storer N, Dogra V, McComsey GA (2010) Heightened inflammation is linked to carotid intima-media thickness and endothelial activation in HIV-infected children. Atherosclerosis 211: 492–498. 20471650 10.1016/j.atherosclerosis.2010.04.008

[19] Ross AC, Rizk N, O'Riordan MA, Dogra V, El-Bejjani D, et al (2009) Relationship between inflammatory markers, endothelial activation markers, and carotid intima-media thickness in HIV-infected patients receiving antiretroviral therapy. Clin Infect Dis 49: 1119–1127. 19712036 10.1086/605578 PMC3895473

[20] Rohani M, Jogestrand T, Ekberg M, van der Linden J, Kallner G, et al (2005) Interrelation between the extent of atherosclerosis in the thoracic aorta, carotid intima-media thickness and the extent of coronary artery disease. Atherosclerosis 179: 311–316. 15777547 10.1016/j.atherosclerosis.2004.10.012

[21] Kablak-Ziembicka A, Tracz W, Przewlocki T, Pieniazek P, Sokolowski A, et al (2004) Association of increased carotid intima-media thickness with the extent of coronary artery disease. Heart 90: 1286–1290. 15486123 10.1136/hrt.2003.025080 PMC1768551

[22] Bots ML, Hoes AW, Koudstaal PJ, Hofman A, Grobbee DE (1997) Common carotid intima-media thickness and risk of stroke and myocardial infarction: the Rotterdam Study. Circulation 96: 1432–1437. 9315528 10.1161/01.cir.96.5.1432

[23] O'Leary DH, Polak JF, Kronmal RA, Manolio TA, Burke GL, et al (1999) Carotid-artery intima and media thickness as a risk factor for myocardial infarction and stroke in older adults. Cardiovascular Health Study Collaborative Research Group. N Engl J Med 340: 14–22. 9878640 10.1056/NEJM199901073400103

[24] Nakanjako D, Kiragga A, Ibrahim F, Castelnuovo B, Kamya MR, et al (2008) Sub-optimal CD4 reconstitution despite viral suppression in an urban cohort on antiretroviral therapy (ART) in sub-Saharan Africa: frequency and clinical significance. AIDS Res Ther 5: 23. 18957083 10.1186/1742-6405-5-23 PMC2605744

[25] Nakanjako D, Ssewanyana I, Nabatanzi R, Kiragga A, Kamya MR, et al (2013) Impaired T-cell proliferation among HAART-treated adults with suboptimal CD4 recovery in an African cohort. BMC Immunol 14: 26. 23786370 10.1186/1471-2172-14-26 PMC3706234

[26] Roman MJ, Naqvi TZ, Gardin JM, Gerhard-Herman M, Jaff M, et al (2006) Clinical application of noninvasive vascular ultrasound in cardiovascular risk stratification: a report from the American Society of Echocardiography and the Society of Vascular Medicine and Biology. J Am Soc Echocardiogr 19: 943–954. 16880089 10.1016/j.echo.2006.04.020

[27] de Groot E, Hovingh GK, Wiegman A, Duriez P, Smit AJ, et al (2004) Measurement of arterial wall thickness as a surrogate marker for atherosclerosis. Circulation 109: III33–38. 15198964 10.1161/01.CIR.0000131516.65699.ba

[28] Parra S, Coll B, Aragones G, Marsillach J, Beltran R, et al (2010) Nonconcordance between subclinical atherosclerosis and the calculated Framingham risk score in HIV-infected patients: relationships with serum markers of oxidation and inflammation. HIV Med 11: 225–231. 19845792 10.1111/j.1468-1293.2009.00766.x

[29] De Socio GV, Martinelli C, Ricci E, Orofino G, Valsecchi L, et al (2010) Relations between cardiovascular risk estimates and subclinical atherosclerosis in naive HIV patients: results from the HERMES study. Int J STD AIDS 21: 267–272. 20378899 10.1258/ijsa.2009.009165

[30] Maggi P, Lillo A, Perilli F, Maserati R, Chirianni A (2004) Colour-Doppler ultrasonography of carotid vessels in patients treated with antiretroviral therapy: a comparative study. AIDS 18: 1023–1028. 15096805 10.1097/00002030-200404300-00010

[31] Fitch KV, Looby SE, Rope A, Eneh P, Hemphill L, et al (2012) Effects of aging and smoking on carotid intima-media thickness in HIV-infection. AIDS 27: 49–57. 10.1097/QAD.0b013e328358b29c PMC3690796 22874518

[32] Mangili A, Polak JF, Skinner SC, Gerrior J, Sheehan H, et al (2011) HIV infection and progression of carotid and coronary atherosclerosis: the CARE study. J Acquir Immune Defic Syndr 58: 148–153. 21792061 10.1097/QAI.0b013e31822d4993

[33] Stein JH, Hsue PY (2012) Inflammation, immune activation, and CVD risk in individuals with HIV infection. JAMA 308: 405–406. 22820794 10.1001/jama.2012.8488

[34] Hsue PY, Lo JC, Franklin A, Bolger AF, Martin JN, et al (2004) Progression of atherosclerosis as assessed by carotid intima-media thickness in patients with HIV infection. Circulation 109: 1603–1608. 15023877 10.1161/01.CIR.0000124480.32233.8A

[35] Currier JS, Kendall MA, Zackin R, Henry WK, Alston-Smith B, et al (2005) Carotid artery intima-media thickness and HIV infection: traditional risk factors overshadow impact of protease inhibitor exposure. AIDS 19: 927–933. 15905673 10.1097/01.aids.0000171406.53737.f9 PMC1373680

[36] Mangili A, Gerrior J, Tang AM, O'Leary DH, Polak JK, et al (2006) Risk of cardiovascular disease in a cohort of HIV-infected adults: a study using carotid intima-media thickness and coronary artery calcium score. Clin Infect Dis 43: 1482–1489. 17083026 10.1086/509575

[37] Dogan S, Duivenvoorden R, Grobbee DE, Kastelein JJ, Shear CL, et al (2010) Completeness of carotid intima media thickness measurements depends on body composition: the RADIANCE 1 and 2 trials. J Atheroscler Thromb 17: 526–535. 20228610 10.5551/jat.3269

[38] Shikuma CM, Ribaudo HJ, Zheng Y, Gulick RM, Meyer WA, et al (2011) Change in high-sensitivity c-reactive protein levels following initiation of efavirenz-based antiretroviral regimens in HIV-infected individuals. AIDS Res Hum Retroviruses 27: 461–468. 20863238 10.1089/aid.2010.0154 PMC3083724

[39] Boger MS, Shintani A, Redhage LA, Mitchell V, Haas DW, et al (2009) Highly sensitive C-reactive protein, body mass index, and serum lipids in HIV-infected persons receiving antiretroviral therapy: a longitudinal study. J Acquir Immune Defic Syndr 52: 480–487. 19911471 10.1097/qai.0b013e3181b939e5 PMC2794651

[40] Masia M, Bernal E, Padilla S, Graells ML, Jarrin I, et al (2007) The role of C-reactive protein as a marker for cardiovascular risk associated with antiretroviral therapy in HIV-infected patients. Atherosclerosis 195: 167–171. 17049532 10.1016/j.atherosclerosis.2006.09.013

[41] Guimaraes MM, Greco DB, Figueiredo SM, Foscolo RB, Oliveira Jr AR, et al (2008) High-sensitivity C-reactive protein levels in HIV-infected patients treated or not with antiretroviral drugs and their correlation with factors related to cardiovascular risk and HIV infection. Atherosclerosis 201: 434–439. 18359028 10.1016/j.atherosclerosis.2008.02.003

[42] Calabro P, Chang DW, Willerson JT, Yeh ET (2005) Release of C-reactive protein in response to inflammatory cytokines by human adipocytes: linking obesity to vascular inflammation. J Am Coll Cardiol 46: 1112–1113. 16168299 10.1016/j.jacc.2005.06.017

